# Estimation of Respiratory Effort Through Diaphragmatic Electromyography Features

**DOI:** 10.3390/s25175463

**Published:** 2025-09-03

**Authors:** Gabriela Grońska, Elisabetta Peri, Xi Long, Sebastiaan Overeem, Johannes van Dijk, Massimo Mischi

**Affiliations:** 1 Department of Electrical Engineering, Eindhoven University of Technology, De Groene Loper 19, 5612AP Eindhoven, The Netherlands; e.peri@tue.nl (E.P.); x.long@tue.nl (X.L.); s.overeem@tue.nl (S.O.); j.p.v.dijk@tue.nl (J.v.D.); m.mischi@tue.nl (M.M.); 2 Center for Sleep Medicine, Kempenhaeghe, Sterkselseweg 65, 5591VE Heeze, The Netherlands

**Keywords:** diaphragmatic electromyography, esophageal pressure, respiratory effort, features, obstructive sleep apnea

## Abstract

Respiratory effort is a critical parameter for assessing respiratory function in various pathological conditions such as obstructive sleep apnea (OSA), as well as in patients undergoing respiratory ventilation. Currently, the gold-standard method for measuring it is esophageal pressure (Pes), which is obtrusive and uncomfortable for patients. An alternative approach is using diaphragmatic electromyography (dEMG), a non-obtrusive method that directly reflects the electrical drive triggering respiratory effort, holding potential for quantifying effort. Despite progress in this area, there is still no clear agreement on the best features for assessing respiratory effort from dEMG. This feasibility study considers several time, frequency, and statistical domain features, providing a comparative analysis to determine their performance in estimating respiratory effort. In particular, we evaluate the correlation of the different features with Pes using overnight recordings from 10 OSA patients and assess their robustness across different signal quality levels with the Kruskal–Wallis test. Our results support that time-domain dEMG features such as the filtered envelope, root mean square, and waveform length (WL) exhibit moderately strong correlations (R > 0.6) with respiratory effort. In terms of robustness to noise, the best features were WL, the area under the curve, and the slope sign change, demonstrating moderately strong to fair correlations (R > 0.5) even in low- to very low-quality signals. In contrast, features like skewness, the mean frequency, and the median frequency performed poorly (R < 0.3), regardless of signal quality, likely because they focus on overall signal characteristics rather than the dynamic and transient changes associated with respiratory effort by temporal features. These findings highlight the importance of selecting optimal features to obtain a reliable estimation of respiratory effort, providing a foundation for future research on non-intrusive methods.

## 1. Introduction

Respiratory effort is a critical parameter for assessing respiratory function in various pathological conditions and clinical settings. Among these, respiratory effort serves as an essential indicator for diagnosis and treatment of obstructive sleep apnea (OSA). OSA is characterized by periodic airway collapse, which leads to increased respiratory effort as the diaphragm progressively exerts more force with each breath until the airway obstruction is resolved or the patient awakens [1]. OSA affects a significant part of the global population, accounting for approximately one billion people worldwide, with prevalence rates exceeding 50% in certain regions [2]. Beyond OSA, respiratory effort is a key parameter in several other conditions, including mechanical ventilation, where it guides ventilatory settings and helps optimize weaning strategies, as well as neuromuscular disorders, where it provides information on respiratory muscle function and disease progression. An accurate assessment of respiratory effort in these conditions is crucial for effective diagnosis and treatment [3].

Currently, the gold standard for assessing respiratory effort across these medical conditions is measuring esophageal pressure (Pes) using a catheter with a pressure sensor inserted into the patient’s esophagus. This sensor detects intrathoracic pressure swings, which increase with greater respiratory effort. However, Pes is measured only in critical cases since the method is intrusive and uncomfortable for patients [4]. Furthermore, its reliability is severely affected by sensor displacement, thoracic volume changes, altered chest wall mechanics, and reduced lung compliance [4,5]. These issues can cause Pes to reflect factors such as lung stiffness or posture effects rather than true respiratory effort. Non-intrusive alternatives like respiratory belts have been proposed, but their measurements are only indirectly linked to effort and are affected by motion artifacts, belt positioning, and patient-specific factors such as body mass index (BMI), which can attenuate respiratory signals.

Surface electromyography (sEMG) can offer a non-intrusive alternative by investigating respiratory muscle electrical activity. During respiration, several muscles are activated, and among these, the diaphragm is responsible for a significant portion of the inhaled volume during both quiet and maximum-effort breathing [6]. Thus, diaphragm electromyography (dEMG) emerges as a direct, non-intrusive method to assess respiratory effort [7]. dEMG has the potential for monitoring respiratory effort, but it comes with several challenges, including electrocardiographic (ECG) contamination, motion artifacts, and environmental noise. Although several methods [4,8,9,10,11,12] have been proposed to mitigate these challenges, in some cases the influence of noise and artifacts cannot be fully eliminated, which can affect signal quality and interpretation.

Previous works proposed different features for the estimation of respiratory effort from dEMG. Most of the existing literature in the field is focused on monitoring patients during mechanical ventilation. In this context, dEMG features such as the variance (VAR) and area under the curve (AUC) of the dEMG envelope have been investigated for their potential to help evaluate diaphragmatic activity as well as enable future optimization of the ventilatory support, preventing diaphragm fatigue and facilitating the weaning protocol [13,14,15].

In the context of sleep monitoring, studies comparing respiratory effort estimated from dEMG with Pes measurements for OSA patients are available. The most promising dEMG features include estimating inspiratory effort based on peak-to-peak (PTP) percentage changes, which have been shown to be statistically comparable to Pes measurements. These findings support the potential of dEMG as a reliable and more comfortable alternative for long-term monitoring [16,17]. However, these studies focused only on the PTP percentage changes, leaving the potential of other dEMG features largely unexplored.

The detection of respiratory effort is also relevant for respiratory task classification. In this context, studies have been conducted to assess and simulate tasks with different levels of respiratory demand. These investigations have explored the ability of dEMG features to distinguish between tasks requiring significant respiratory effort and those involving minimal or no effort. Among the more common features proposed in this context are the AUC, mean absolute value (MAV), and VAR [18,19,20,21,22].

Beyond estimating respiratory effort, dEMG has also been proposed for the classification and recognition of breathing patterns. Analyses of dEMG features such as the root mean square (RMS), the mean frequency (MNF), and others have shown that it is possible to distinguish between different respiratory states, improving the understanding of both normal and altered breathing patterns [23,24,25,26,27].

Despite advancements in respiratory monitoring, a comprehensive performance comparison of different dEMG features for assessing respiratory effort is still missing, and no consensus has been reached regarding an optimal feature set. While various features have been explored in fields such as mechanical ventilation and respiratory pattern recognition, their effectiveness in accurately estimating respiratory effort from dEMG remains unclear. Additionally, several features commonly used in fields that are not directly related to respiration but still closely related to effort estimation from the electrical activity of muscles are underexplored. This applies to well-known features proposed for sEMG-based estimation of muscle force, fatigue, and task classification such as total power (TTP) and Willison amplitude (WAMP), among others, which, despite their potential relevance, have never been specifically investigated for respiratory effort. These features have been extensively studied in other contexts to characterize signal properties and improve the understanding of muscle dynamics [28,29,30,31]. They have also proven effective in identifying distinct muscle activities and evaluating various levels of fatigue [32,33,34,35]. Exploring these less-studied features in the context of dEMG and respiration could provide new insights into respiratory effort assessment, particularly since the force generated by the diaphragm plays a central role in inspiratory effort, and muscle fatigue can directly impair this force. As such, features responsive to muscle force and fatigue may offer complementary value in capturing the physiological basis of respiratory effort.

This paper presents a feasibility study that aims to address these gaps by extracting and comparing standard dEMG features from fields like mechanical ventilation and sleep respiratory monitoring, alongside additional features originally proposed for assessing electromyographic activity. The evaluation is conducted using data from 10 OSA patients during overnight recordings, comparing the dEMG features against the esophageal Pes signal. By analyzing how well these features, extracted from different dEMG signal quality segments, correlate with Pes measurements, we aim to identify those that accurately capture respiratory effort and remain reliable even with noisy or low-quality signals. This work provides a foundation for standardizing dEMG-based respiratory effort assessments and highlights the most effective features for clinical and research applications.

## 2. Materials and Methods

### 2.1. Dataset

**Figure 1:**
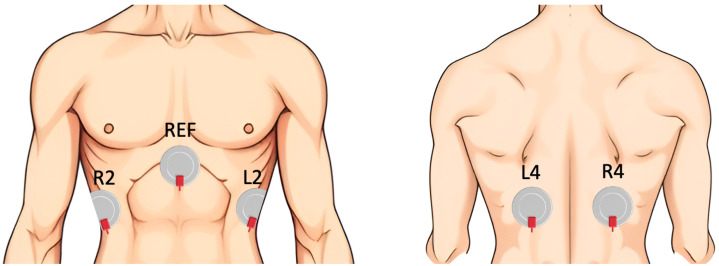
Sensor placement for dEMG measurement.

The analysis was based on 10 patients from the SOMNIA dataset, acquired at the Kempenhaeghe Sleep Disorder Center [36]. This study was approved by the medical ethical committee of the Máxima Medical Center (Veldhoven, The Netherlands, File no: N16.074), and all patients gave written informed consent. The research was conducted in accordance with the ethical standards outlined in the Declaration of Helsinki. Access to the fully anonymized data for this study was granted on 7 July 2022. Out of the 66 patients with OSA who underwent full-night polysomnography, 10 were selected based on the availability of the reference signal (Pes), ensuring a reliable ground truth for the evaluation of the features. This paper focuses on the analysis of Pes and dEMG signals. The Pes signal was acquired through a flexible catheter equipped with a pressure sensor at its tip, which was positioned transnasally in the esophagus above the diaphragm (Gaeltec CTO-1, sampling frequency = 128 Hz). Four dEMG monopolar channels were recorded using Ag/AgCl electrodes (H34SG) located on the 6th intercostal space (two on the frontal and two on the dorsal diaphragm) and one reference electrode on the sternum (sampling frequency = 512 Hz), as shown in Figure 1.

### 2.2. Signal Preprocessing

To explore the relationship between respiratory effort determined from Pes and dEMG, as well as to extract the investigated features, the signals were preprocessed. The Pes signal was up-sampled from 128 Hz to 512 Hz to align with the dEMG sampling rate. Subsequently, a fourth-order band-pass Butterworth filter was applied within the respiratory frequency range of 0.05 to 0.6 Hz to derive the Pes envelope.

For the dEMG signal, noise reduction was achieved using a fourth-order Butterworth filter with a band-pass range of 5 to 250 Hz [37], along with a 50 Hz notch filter to eliminate power-line interference. The bipolar configuration was obtained by subtracting signals from neighboring electrodes (L2–L4 as channel 1, L4–R4 as channel 2, and R2–R4 as channel 3), resulting in three channels. Significant interferences from the electrocardiogram signal and movement artifacts were mitigated using a recently developed SVD-based algorithm [38], which showed superior performance with respect to the state of the art [10].

### 2.3. Quality Assessment

The quality of the preprocessed dEMG signals was assessed. To that end, the dEMG envelope was computed using a third-order low-pass Butterworth filter with a 0.6 Hz cut-off frequency. The cut-off frequency was chosen to match the low-frequency components of the dEMG signal, which corresponds to respiration [39]. The entropy, frequency spectrum, and frequency density around the respiratory values of the dEMG envelope were computed on windows with a length of 60 s and an 80 % overlap. For each window, a quality index (QI) was computed as

(1) QI=mean(QE+QS+QD),

where QE represents the percentage ratio between the signal’s entropy and the entropy of a random signal, QS is a fixed percentage determined by the peak of the power spectral density, and QD represents the percentage ratio of the power concentrated around the respiratory frequency to the total power within the entire window. The dEMG signal was divided into four quality intervals: very low quality: 0–50%; low quality: 50–75%; high quality: 75–90%; and very high quality: 90–100%. This division was based on the number of affected factors in the QI: in the very low-quality range, all three factors were impacted; in the low-quality range, two factors were affected; in the high-quality range, only one factor was influenced; and in the very high-quality range, the impact on the factors was minimal. The channel with the largest amount of signal with quality > 75% was chosen for the analysis and used to calculate all the features.

The same QI was used for Pes. To guarantee high-quality reference data, only windows with a Pes QI > 75% were included in the analysis.

### 2.4. Feature Estimation

An extensive literature review showed that various features were used to assess respiratory effort from electromyographic signals. For the purpose of this paper, features from the following fields were considered: respiration patterns detection (RPD), respiratory effort (RE) detection/assessment, mechanical ventilation (MV), and estimation of fatigue, movement, and effort (FME) through skeletal muscles.

An overview of the features proposed in the literature is reported in Table 1. Among them, a total of 16 features (in bold) were selected. The filtered envelope (FENV), RMS, AUC, and PTP were chosen for comparative analysis, as they are the most frequently and consistently used in the dEMG context. Of the remaining 19 features, 12 were retained to avoid redundancy and improve interpretability. For time-domain measures of the signal envelope, sample entropy, MAV, and VAR were chosen, replacing related metrics such as mean amplitude, standard deviation, and mean absolute deviation that carry highly correlated information; additionally, waveform length (WL), WAMP, slope sign change (SSC), and zero crossing (ZC) were included due to their ability to characterize signal complexity and dynamics. In the statistical domain, skewness (SKEW) and kurtosis (KURT) were retained to capture distribution asymmetry. In the frequency domain, MNF and median frequency (MDF) were selected for their robustness, widespread use, and complementary representation of spectral content, while TTP was chosen over the simple square integral as its frequency-domain equivalent. The selected subset captures complementary aspects of the signal while minimizing redundancy in the feature set. All selected features are detailed in the following sections, categorized into time-domain, statistical-domain, and frequency-domain features.

**Figure 2:**
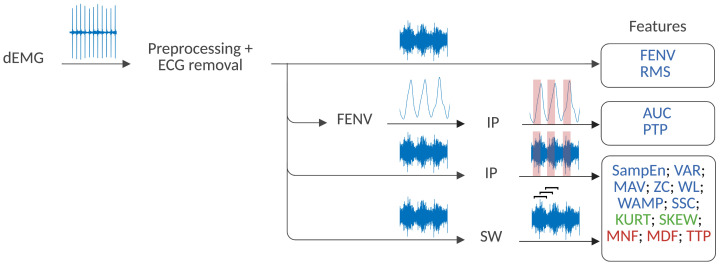
Strategy for dEMG feature extraction. Time-domain features are highlighted in blue, statistical-domain features in green, and frequency-domain features in red. The SW and IP stand for the sliding window and the inhalation phase strategy, respectively.

**Figure 3:**
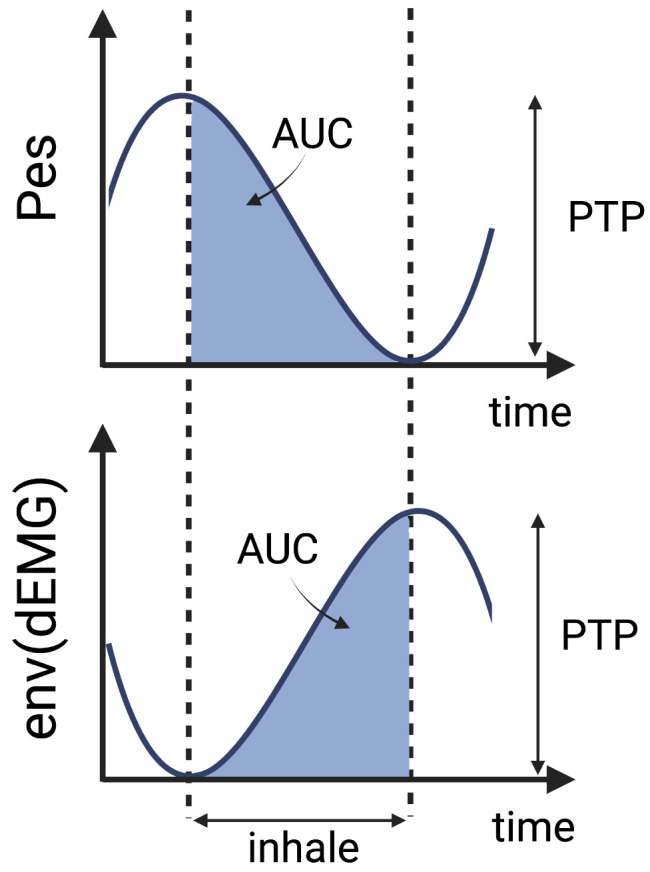
Schematic representation of the principle behind AUC and PTP extraction.

The analysis of all the features, with the exception of FENV and RMS, was performed following two different strategies, as presented in Figure 2. Firstly, an analysis of the inhalation phase (IP) was performed. To that end, inspiratory windows were used to calculate each feature. The inspiratory phase was defined using Pes as the time between the maximum Pes value and the following minimum (see Figure 3). In this case, the window varied depending on the duration of the inspiratory phase. Secondly, a sliding window (SW) approach was applied, with features computed using a fixed window of 250 ms (128 samples) and a 90% overlap to ensure consistent and comparable results across the signal. Previous studies support the choice of a 250 ms window, as windows smaller than 300 ms are commonly used in the literature to maintain a balance between accurate feature extraction and computational efficiency [24,30,41,47,49].

#### 2.4.1. Time Domain

Time-domain features are among the most widely used for analyzing both the Pes and dEMG signals across various applications. In this study, the FENV [19,40] and the RMS envelope [14,19,20,21,24,29,30,31,32,33,34,41,42,43], both derived from the rectified preprocessed dEMG signal, were included. The FENV was obtained using a third-order low-pass Butterworth filter with a 0.6 Hz cut-off frequency. The RMS envelope was derived using a sliding window of *N* samples covering 1 s with a 90% overlap as

(2) $$\mathrm{RMS}=\sqrt{\frac{1}{N}\sum_{n=1}^{N}s^{2}\left(n\right)}\;$$

where s(n) is the *n*-th sample of the dEMG signal.

To capture amplitude characteristics, the AUC [14,15,21,23,25,44] and PTP values [16,17,27,34,44,45] were calculated during the inspiratory phase of the FENV signal. The start of expiration was identified by minimal pressure and peak electrical activity, following the Pes-based definitions of inspiratory phase boundaries according to the state-of-the-art methods reported in previous studies [13,16,17]. This allowed for the calculation of the AUC during inhalation and the estimation of PTP as the amplitude difference between exhalation and inhalation, as illustrated in Figure 3.

Furthermore, other time-domain features were obtained from the preprocessed dEMG. These features have been proposed in various applications, such as estimating effort in mechanical ventilation or classifying tasks and assessing fatigue in skeletal muscle studies. In the following section, we refer to *n* indicating the *n*-th sample of the time series, and to *N* indicating the window length, corresponding to the two aforementioned strategies, SW and IP.

Among the features considered, SampEn quantifies the irregularity of lower-quality physiological time series [26,41,46,47]. Its computation followed the method described in [46], with m=1, r=0.3·std, and std indicating the signal’s standard deviation.

The mean absolute value (MAV) represents the average absolute value of the EMG signal, reflecting the overall muscle activation level [24,29,30,31,33,35]. It is given as

(3) $$\mathrm{MAV}=\frac{1}{N}\sum_{n=1}^{N}\left|s\left(n\right)\right|\;.$$

Variance (VAR) quantifies the variability of the EMG signal, which can indicate changes in muscle activity [14,15,28,31]. It is formulated as

(4) $$\mathrm{VAR}=\frac{1}{N-1}\sum_{n=1}^{N}{\left(s\left(n\right)-\mu \right)}^{2}\;,$$

where μ corresponds to the mean value of all samples in the considered window. Waveform length (WL) is a measure of the complexity of the EMG signal. It is defined as the cumulative length of the EMG waveform over the time segment [24,28,29,31], and it is given as

(5) $$\mathrm{WL}=\sum_{n=1}^{N-1}\left|s\left(n+1\right)-s\left(n\right)\right|\;.$$

Willison amplitude (WAMP) is instead formulated as

(6) $$\begin{array}{cc} \mathrm{WAMP} & =\sum_{n=1}^{N-1}[f(|s\left(n\right)-s\left(n+1\right)|)], \end{array}$$

(7) $$\begin{array}{cc} f(x) & =\left\{\begin{array}{cc} 1, & \mathrm{if}\;x\geq \;\mathrm{th}\\ 0, & \mathrm{otherwise}, \end{array}\right. \end{array}$$

indicating a threshold that is set to the root mean square of the signal considered [49,50].

Slope sign change (SSC) counts the number of changes in the slope of the EMG signal, indicating muscle contraction dynamics as [24,28,30,31]

(8) $$\begin{array}{c} \mathrm{SSC}=\sum_{n=2}^{N-1}\left[f\left(\left|(s(n)-s(n-1))\times (s(n)-s(n+1))\right|\right)\right], \end{array}$$

(9) $$\begin{array}{c} f\left[x\right]=\left\{\begin{array}{cc} 1, & \mathrm{if}\;x\geq \mathrm{th}\\ 0, & \mathrm{otherwise}. \end{array}\right. \end{array}$$

Zero crossing (ZC) is a measure of the frequency information of the EMG signal defined in the time domain. It counts the number of times the EMG amplitude crosses zero within a certain period, which indicates muscle fiber activation patterns. To prevent the influence of low-voltage fluctuations or background noise, a threshold condition is applied [20,24,28,31,33]. The calculation is defined as

(10) $$\begin{array}{c} \mathrm{ZC}=\sum_{n=1}^{N-1}\left[\mathrm{sgn}\left(s\left(n\right)\times s\left(n+1\right)\right)\cap \left|s(n)-s(n+1)\right|\geq \mathrm{th}\right], \end{array}$$

(11) $$\begin{array}{c} \mathrm{sgn}\left(x\right)=\left\{\begin{array}{cc} 1, & \mathrm{if}\;x\geq \mathrm{th}\\ 0, & \mathrm{otherwise}. \end{array}\right. \end{array}$$

#### 2.4.2. Statistical Domain

Kurtosis (KURT) and skewness (SKEW) are statistical measures that describe the distribution shape of the EMG signal, providing information on signal characteristics and potential abnormalities [24,28]. They are given as

(12) $$\mathrm{KURT}=\frac{\frac{1}{N}\sum_{n=1}^{N}{\left(s\left(n\right)-\mu \right)}^{4}}{\sigma^{4}},\;$$

(13) $$\mathrm{SKEW}=\frac{\frac{1}{N}\sum_{n=1}^{N}{\left(s\left(n\right)-\mu \right)}^{3}}{\sigma^{3}},\;$$

with μ and σ corresponding to the mean and standard deviation of the distribution. These features, similar to the others, were also computed using the two strategies mentioned above; where window length *N* was equal to the inhalation phase and the sliding window, respectively.

#### 2.4.3. Frequency Domain

Frequency-domain features were selected to help in assessing muscle fatigue and effort by indicating shifts in the frequency content of the EMG signal. The window of *N* samples used across the equations, which determines the length of the frequency bin, was considered using two separate strategies. Firstly, the inhalation phase, and secondly, the sliding window.

The mean power frequency (MNF) represents the average power spectral frequency of the EMG signal and is calculated by dividing the weighted sum of the power spectrum and corresponding frequencies by the total intensity of the power spectrum [19,20,26,28,29,30,32,34,35,48]. It can be calculated as

(14) $$\mathrm{MNF}=\frac{\sum_{j=1}^{N}f_{j}P_{j}}{\sum_{j=1}^{N}P_{j}},\;$$

where *f_j_* is the frequency of the spectrum at frequency bin *j* and *P_j_* is the EMG power spectrum at frequency bin *j*.

The median power frequency (MDF) is the frequency that divides the power spectrum into two halves. Essentially, MDF represents the midpoint of the total power feature (TTP) [21,28,29,30,32,34,35], which is given as

(15) $$\mathrm{MDF}=\sum_{j=1}^{\mathrm{MDF}}P_{j}=\sum_{j=\mathrm{MDF}}^{N}P_{j}=\frac{1}{2}\sum_{j=1}^{N}P_{j}.\;$$

Total power (TTP) is the sum of the EMG power spectrum and is often referred to as the zero spectral moment or energy [30]. It is defined as

(16) $$\mathrm{TTP}=\sum_{j=1}^{N}P_{j}=\mathrm{SM}0.$$

### 2.5. Performance Assessment

The performance of each dEMG feature in estimating respiratory effort was assessed by comparison with Pes, the gold standard. The Spearman correlation coefficient (R) was chosen as a performance metric to evaluate how well each feature tracked the development of respiratory effort over time. The correlation strength was classified as poor (R < 0.3), fair (0.3 < R < 0.6), moderately strong (0.6 < R < 0.8), and very strong (R > 0.8) [51].

Depending on whether a corresponding feature could be extracted from the Pes signal, different comparison approaches were adopted. The FENV, RMS, AUC, and PTP were computed for both dEMG and Pes, allowing their direct comparison through correlation. The other features could not be directly computed on the Pes signal, and as such, the comparison between dEMG features and Pes was performed using two alternative strategies. The features computed for the inspiratory phase (IP) were validated against the AUC of Pes, representing an estimation of the effort during the inhalation phase. Furthermore, the features obtained using a sliding window (SW) approach were compared with the mean value of the Pes envelope over the same window. This approach ensured that all dEMG features, whether directly or indirectly linked to Pes, could be evaluated consistently.

The described approach was first applied only to signals with a quality index above 90%, representing the optimal signal conditions. Subsequently, only a subset of features was chosen for further investigation across varying signal qualities. This subset included features that demonstrated strong performance in high-quality signals (correlation R > 0.5), as well as the most commonly used features proposed in the literature for dEMG (FENV, RMS, AUC, and PTP). In this way, only features with a consistent and interpretable relationship to respiratory effort were tested under more challenging, noisier conditions.

To assess feature reliability across different signal quality levels, the correlation analysis was repeated for very low- (0–50%), low- (50–75%), and high-quality (75–90%) signals, then compared with the results from very high-quality signals (>90%). Statistical differences across quality levels were tested using the Kruskal-Wallis multi-sample test, followed by the Mann-Whitney Bonferroni-corrected post-hoc test. This approach allowed for the evaluation of feature performance across different signal quality levels, offering insights into their robustness under less optimal conditions.

## 3. Results

The results of 10 patients with an average age of 46 ± 12 and a BMI of 28 ± 3 were analyzed and presented first for the highest signal quality and then for all quality intervals. The features were computed based on the optimal channel, as explained in the Quality Assessment Section. The performance of the quality index (QI) used for signal selection was visually inspected by an expert and considered acceptable for this analysis. This led to the selection of channels 2 and 3 in 40% of the cases each, while channel 1 was identified as the best for 20% of the patients. The dataset, including ∼ 83 h of recordings, was then divided into four quality intervals: very low (VL) for 0% < QI < 50%, 50% < QI < 75% for low (L), 75% < QI < 90% for high (H), and very high (VH) for QI > 90%. These intervals included ∼10% (∼8 h), ∼2% (∼2 h), ∼19% (∼16 h), and ∼65% (∼54 h) of the signal for VL, L, H, and VH, respectively.

**Figure 4:**
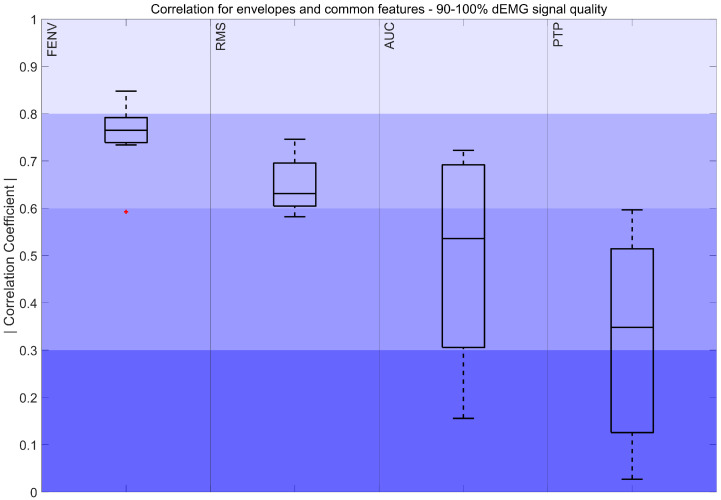
Correlation coefficient of the FENV, RMS, AUC, and PTP for the 90–100% interval. The color intensity presents the strength of the correlation, ranging from poor (0–0.3) to fair (0.3–0.5), moderately strong (0.6–0.8), and very strong (0.8–1). The red marker indicates outliers.

**Figure 5:**
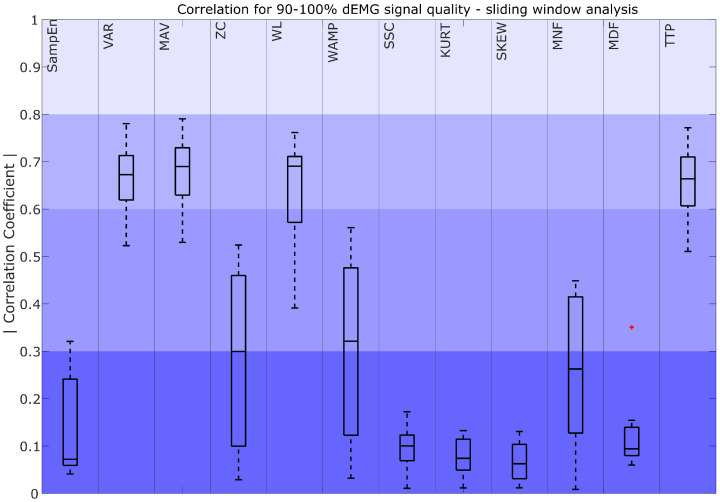
Correlation coefficient for 90–100% dEMG signal quality in the sliding window approach. The color intensity presents the strength of the correlation, ranging from poor (0–0.3) as the most saturated to fair (0.3–0.5), moderately strong (0.6–0.8), and very strong as the least saturated (0.8–1). The red plus signs indicate outliers.

**Figure 6:**
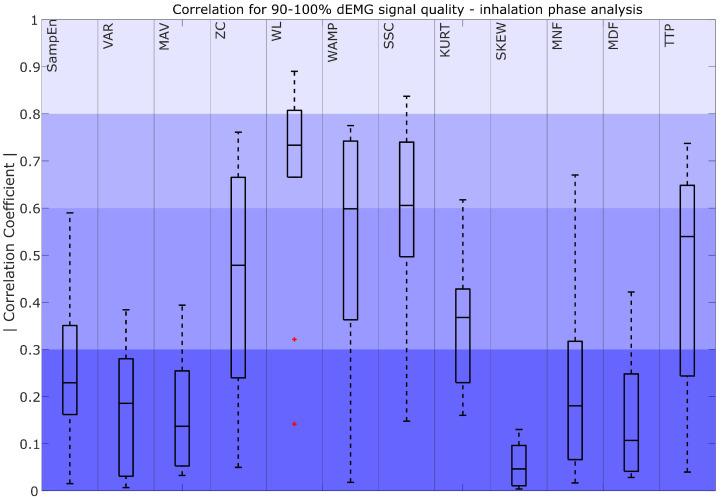
Correlation coefficient for 90–100% dEMG signal quality in the inhalation phase. The color intensity presents the strength of the correlation as poor (0–0.3) as the most saturated, fair (0.3–0.5), moderately strong (0.6–0.8), and very strong as the least saturated (0.8–1). The red plus signs indicate outliers.

The absolute values of the correlation coefficients for features derived from signals with quality exceeding 90% are shown in Figure 4, Figure 5 and Figure 6

Figure 4 demonstrates a moderately strong correlation between the FENV, RMS, and Pes envelope. Additionally, AUCs of Pes and dEMG obtained a moderately strong to fair correlation, while the correlation between PTP values ranged from fair to poor. Both features presented high variability in the results.

A comparison between dEMG features computed with the sliding window (SW) approach and Pes is presented in Figure 5. The results show that VAR_SW_, MAV_SW_, WL_SW_, and TTP_SW_ obtained moderately strong correlations. For ZC_SW_, WAMP_SW_, and MNF_SW_, correlations ranged from fair to poor, with high variability. All other characteristics (SampEn_SW_, SSC_SW_, KURT_SW_, SKEW_SW_, and MDF_SW_) obtained poor correlations.

Figure 6 compares the dEMG features computed in the inhalation phase with the AUC of Pes. The results show that WL_IP_ achieved moderate to very strong correlations. WAMP_IP_ and SSC_IP_ obtained moderately strong median correlations, with the interquartile range of the coefficients spanning from moderately strong to fair, while TTP_IP_ and ZC_IP_ demonstrated a range of poor to moderately strong correlations with a median value remaining fair. Additionally, KURT_IP_ exhibited a fair to poor correlation. The remaining features, such as SampEn_IP_, VAR_IP_, MAV_IP_, SKEW_IP_, MNF_IP_, and MDF_IP_, showed a poor correlation with the AUC of Pes.

**Figure 7:**
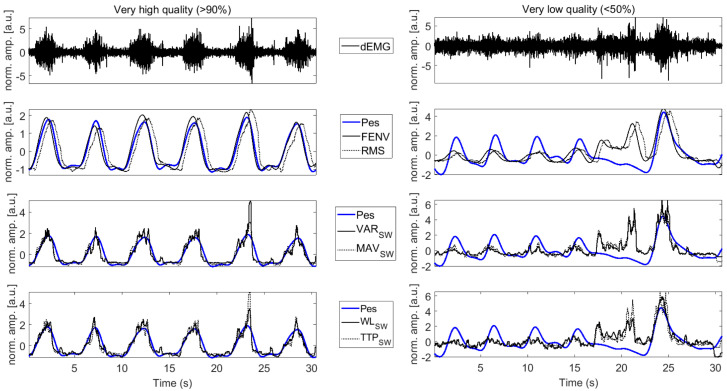
The dEMG signal and a selection of related features for a very high-quality dEMG signal (**left column**) and a very low-quality dEMG signal (**right column**). The gold-standard respiratory effort estimated with Pes is reported in blue. dEMG signals reported in the top panels are preprocessed to remove ECG contamination. dEMG features are reported in solid and dotted black lines. Amplitude is normalized to improve readability.

The standard dEMG features (FENV, RMS, AUC, and PTP), as well as features performing better at high signal quality (i.e., showing median correlation R > 0.5), were selected for further evaluation across different quality intervals. A representative example of VH and VL quality signals across selected features is presented in Figure 7.

**Figure 8:**
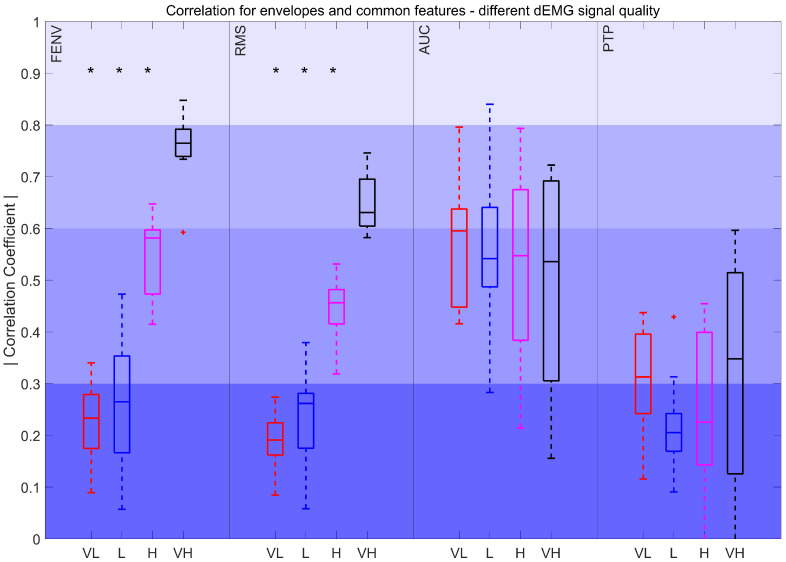
Correlation coefficient for FENV, RMS, AUC, and PTP for different intervals. Very low (VL): 0%<QI<50%, Low (L): 50<QI<75%, High (H): 75<QI<90%, and Very high (VH): 90<QI<100%. The red plus signs indicate outliers. *: *p* < 0.017

Figure 8 illustrates a decrease in the correlation for FENV and RMS, with correlations dropping from moderately strong to poor as the quality of the signal declines. Statistically significant differences were observed between very low-, low-, and high-quality signals when compared to very high-quality signals. In contrast, AUC and PTP showed a less pronounced drop in correlation, and these differences were not statistically significant. For the AUC, the correlation remained fair and moderately strong across intervals, whereas the fair correlation obtained through PTP dropped to poor levels as signal quality worsened.

**Figure 9:**
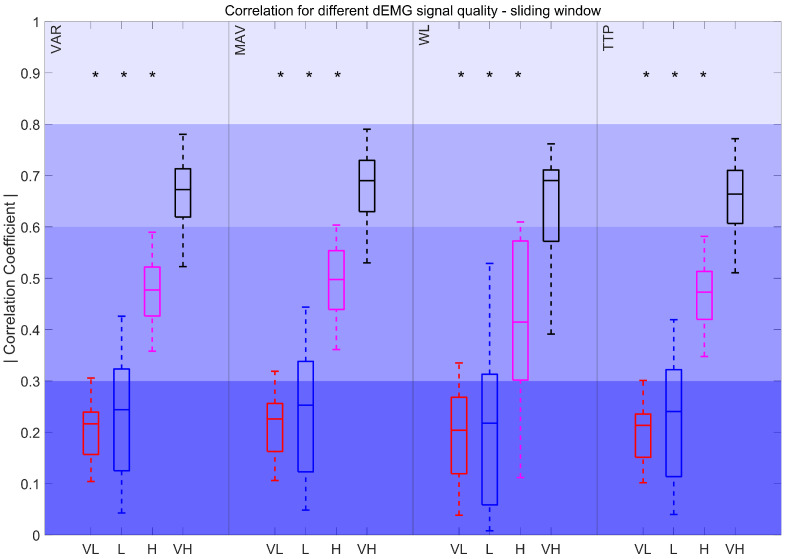
Correlation coefficient for different dEMG signal quality levels in the sliding window approach. Very low (VL): 0%<QI<50%, Low (L): 50<QI<75%, High (H): 75<QI<90%, and Very high (VH): 90<QI<100%. *: *p* < 0.017

The correlation coefficients for features derived using sliding windows, compared to the resampled Pes envelope, are depicted in Figure 9. The results show significant differences between VL, L, and H quality intervals when compared to VH quality signals. The correlations for VAR_SW_, MAV_SW_, WL_SW_, and TTP_SW_ declined from moderately strong to poor as signal quality decreased.

**Figure 10:**
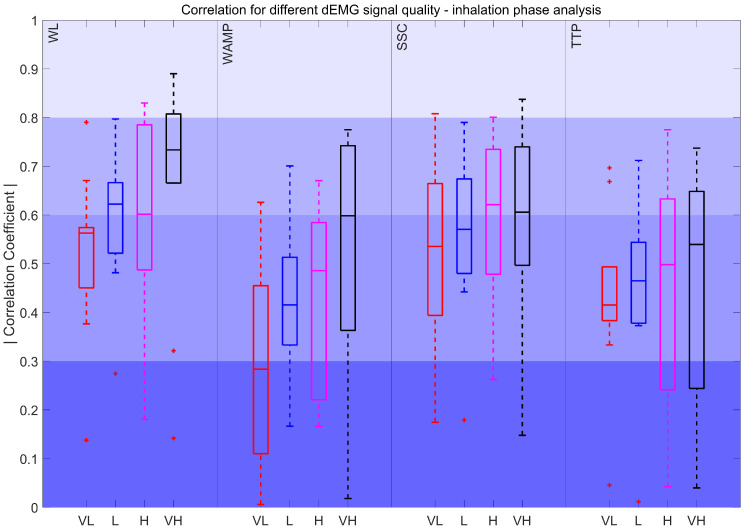
Correlation coefficient for different dEMG signal quality levels in the inhalation phase. Very low (VL): 0%<QI<50%, Low (L): 50<QI<75%, High (H): 75<QI<90%, and Very high (VH): 90<QI<100%. The red plus signs indicate outliers.

Lastly, Figure 10 illustrates the correlation of the Pes AUC with the features derived from the inspiratory phase across all quality intervals. The features did not show statistically significant variations between intervals. WL_IP_, SSC_IP_, and TTP_IP_ features consistently maintained correlations within the fair to moderately strong range. For WAMP_IP_ the correlation dropped to poor in lower-quality signals.

## 4. Discussion

This study tackles the challenge of investigating the performance of various dEMG features to assess respiratory effort. Although some research has been performed in different fields, including sleep monitoring and mechanical ventilation, there is still a lack of agreement on the most effective features for respiratory effort monitoring. By comparing the performance of state-of-the-art features in estimating respiratory effort, this work highlights the most promising options and the key challenges that remain.

First, the results obtained for the highest signal quality (90–100%) were analyzed. Considering the most common features, FENV and RMS stood out for their strong and consistent correlations (R > 0.6) with Pes signals. This suggests that, when the signal quality is high, these features are reliable for capturing respiratory effort dynamics. Furthermore, we also considered the AUC and PTP, as they are among the most widely used features in the literature [16,17,44,45]. The results showed that, despite both AUC and PTP obtaining a median fair correlation, the AUC consistently outperforms PTP. While a direct comparison of the AUC and PTP for estimating respiratory effort in dEMG is not available in the literature, these findings align with skeletal muscle surface EMG studies, where integrated EMG was found to be less affected by transient noise and movement-related fluctuations than peak-based measures [52]. Consistent with these observations, the weaker performance of PTP in our study likely stems from the sensitivity to artifacts. Since it relies on the maximum value, it can be easily skewed by outliers or residual artifacts, reducing its accuracy for estimating respiratory effort and suggesting that PTP may not be robust even with high-quality dEMG signals.

Considering more advanced features, our findings show that the strategy used to compute the feature, i.e., the sliding window (SW) or the inspiratory phase window (IP), has an impact on the selection of the best-performing features. For the SW approach, features such as VAR_SW_, MAV_SW_, WL_SW_, and TTP_SW_ had moderately strong correlations (R > 0.6). These features effectively capture patterns and characteristics that correspond to the dEMG envelope. Notably, VAR_SW_, MAV_SW_, and WL_SW_ are also frequently used in previous surface EMG studies as common descriptors of muscle activation patterns [30]. However, in the IP approach, features such as WL_IP_, WAMP_IP_, and SSC_IP_ stood out with moderately strong median correlations, while TTP_IP_ showed a median fair correlation with R > 0.5. In the case of IP analysis, the focus shifts to evaluating the features that are most capable of representing the inhalation phase. The differences in the selection of well-performing features come from the distinct roles of these strategies. Consequently, features that excel in describing the overall respiratory effort described through the Pes envelope may not necessarily correspond with the best indicators of the inspiratory phase, and vice versa.

The results reported in our work also revealed a large impact of signal quality on the performance of the mentioned features. Specifically, when the evaluation extended to lower-quality intervals, the performance of many features dropped significantly. This highlights the challenge of choosing the best feature that is robust in noisy or degraded signals, which is a common issue in clinical and real-world settings. For example, FENV and RMS showed large differences in the correlation strength between high- and low-quality signals, as seen in Figure 7 and Figure 8. Interestingly, the AUC was less affected by signal quality degradation, suggesting that it could have broader applications in situations where dEMG data may not always be optimal. PTP also maintained a stable performance over different signal quality levels. However, it is important to note that PTP showed poor correlations for half of the quality intervals in contrast to the AUC, which consistently outperformed PTP, reinforcing its reliability and potential as a more robust feature for assessing respiratory effort.

Focusing on the dEMG-specific features performing well with high-quality signals in sliding window analysis, such as VAR_SW_, MAV_SW_, WL_SW_, and TTP_SW_, their reliability dropped sharply in lower-quality signals (Figure 5, Figure 7 and Figure 9). This behavior closely follows the trends observed for envelopes derived from FENV and RMS since the time evolution of dEMG is ultimately the core signal compared across features. The performance drop might be caused by the fact that the SW analyzes the entire signal, including the inhalation and exhalation phases. This poses a challenge for dEMG-based analysis since exhalation is driven by the pressure gradient, while the respiratory muscles are mostly inactive. However, the Pes signal captures the actual pressure changes, making it inherently more informative during the exhalation phase. This fundamental difference explains why features that use the entire dEMG envelope may exhibit a less predictable relationship compared to features analyzed during active breathing phases, especially when considering different signal quality levels.

On the other hand, the features derived from IP analysis, such as WL_IP_, WAMP_IP_, SSC_IP_, and TTP_IP_, correlated well with the Pes AUC in high-quality intervals and maintained reliability across different quality levels (Figure 10). This might be caused, as mentioned before, by focusing on the active phase of the respiratory effort. Among these features, WL achieved the highest values for all signals of different quality, as it better reflects the intensity and duration of muscle activity, which aligns more closely with the respiratory effort captured by Pes during the active breathing phases. SSC_IP_ was the second-best-performing feature, consistently maintaining a median correlation above 0.5 across quality levels, likely due to its ability to capture structural changes in the signal, which are associated with inspiratory muscle activation. However, WAMP_IP_ showed the poorest performance in lower-quality signals (R < 0.3), likely due to its sensitivity to noise and signal irregularities. Since it relies on precise detection of amplitude changes, it is more prone to distortion, highlighting its strong dependence on signal quality.

Additionally, it is important to note that statistical features, such as MNF and MDF, performed poorly (R < 0.3) regardless of the noise level and the calculation strategy used. This may be due to the fact that these features primarily capture overall signal characteristics such as frequency distribution or statistical moments, which can be less sensitive to dynamic and transient changes in respiratory effort. Although using only high-quality signals for their calculation, these features may not adequately reflect the specific muscle activity associated with respiratory effort, as they do not account for more localized, time-dependent variations in the dEMG signal.

Among the features examined, several commonly used metrics such as FENV, RMS, and AUC demonstrated consistent relevance, confirming their established utility in dEMG analysis. Notably, features less explored in this context, such as WL_IP_, SSC_IP_, VAR_SW_, MAV_SW_, and TTP_SW_, also showed promising performance. Overall, the AUC, WL_IP_, and SSC_IP_ calculated during the inhalation phase emerged as the most robust features, with WL_IP_ obtaining the highest correlation values. For higher-quality signals, FENV achieved the highest correlation results, proving to be the most effective at representing the dEMG envelope. Furthermore, RMS and features calculated using the sliding window strategy, such as VAR_SW_, MAV_SW_, WL_SW_, and TTP_SW_, also demonstrated strong performance in high-quality conditions. However, despite its common use, the PTP feature did not perform well in this analysis, suggesting that it may be less suitable for this application, possibly due to its sensitivity to artifacts.

These observations should also be considered in the context of the studied population. Our evaluation focused on OSA patients, where exerted respiratory effort spans the full physiological range, from normal breathing patterns to progressively higher efforts during obstructive events, which are known to follow a crescendo pattern [53,54]. Given the prolonged overnight recordings and the wide spectrum of respiratory patterns captured, the findings may be transferable not only to healthy populations but also to patients with clinical conditions where respiratory effort is a critical factor for monitoring and diagnosis, such as COPD, neuromuscular disorders, or mechanical ventilation. In these conditions, where the reliable assessment of respiratory effort is often challenging, robust features such as the AUC, WL_IP_, and SSC_IP_, which demonstrated consistent performance across varying signal qualities, may provide a practical advantage. Together, these aspects strengthen the potential clinical utility of the identified features and support their use in future studies exploring respiratory monitoring across diverse patient groups.

### 4.1. Recommendations

Given these findings, in cases where high-quality signals are available, FENV, RMS, AUC, WL, TTP, VAR_SW_, MAV_SW_, ZC_IP_, WAMP_IP_, and SSC_IP_ are highly recommended due to their good performance and reliability. On the other hand, in real-life scenarios where noisy signals are more common, features, such as the AUC, WL_IP_, and SSC_IP_, obtained from the inspiratory phase are best suited since they maintain robustness across different signal qualities.

### 4.2. Limitations and Future Research

While this study offers insights into dEMG features, there are a few limitations to consider. A key limitation is that the dataset included recordings from only 10 subjects. This may reduce the generalizability of the findings, as inter-subjects variability could influence respiratory dEMG characteristics. In addition, the distribution of events may not fully capture the balance between apneas and hypopneas, as some patients were more prone to hypopneic episodes. Nevertheless, the recordings covered the entire nights (6 to 8 h per patient), capturing a variety of respiratory patterns associated with sleep apnea, thus creating a representative dataset for the feasibility study. With this foundation, future work should expand to larger and more diverse populations to confirm and extend the findings. Looking ahead, further research could focus on developing novel and robust features, such as those based on multichannel analysis, to improve the accuracy and reliability of the estimation of respiratory effort derived from dEMG. Furthermore, investigation into the respiratory phase for dEMG could prove useful for improvements in capturing inspiration onset and offset, as mentioned in [55]. Although the best channel was selected based on the QI, further studies could explore the optimal channel or channel configuration. To further enhance feature performance and overcome the limitations of individual features, future work could also integrated the identified features into a machine learning framework aimed at regressing the respiratory effort while integrating the different signal characteristics carried by each feature. Finally, the findings of this study could be applied to the detection and classification of respiratory events in sleep disorders, such as sleep apnea, where the identified features, particularly those robust across different signal qualities, could support improved monitoring and diagnosis despite variations in signal quality.

## 5. Conclusions

In conclusion, this paper demonstrates the relevance of an appropriate choice of features to estimate respiratory effort under varying conditions and provides recommendations on the most appropriate feature in different signal quality conditions. Specifically, the AUC, WL_IP_, and SSC_IP_ calculated during the inhalation phase emerged as the most robust features overall, with WL_IP_ showing the highest correlation with Pes. In higher-quality signals, FENV was the top-performing feature, while RMS and several sliding window measures (VAR_SW_, MAV_SW_, WL_SW_, and TTP_SW_) also achieved strong performance. In contrast, the widely used PTP feature underperformed, suggesting its limited suitability for respiratory effort estimation in this context. This work provides a solid foundation for advancing non-invasive methods for respiratory effort estimation in clinical practice and supports further investigation through non-invasive monitoring of sleep-related breathing problems.

## Figures and Tables

**Table 1:** Features overview from different fields. The features selected for the comparative analysis are highlighted in bold.

Feature	Acronym	Field	Included Studies
Time domain			
Filtered Envelope	**FENV**	RE	[19,40]
Root Mean Square	**RMS**	MV, RE, and FME	[14,19,20,21,24,29,30,31,32,33,34,41,42,43]
Area Under The Curve	**AUC**	MV, RPD, and RE	[14,15,21,23,25,44]
Peak-to-Peak/Inspiratory Effort	**PTP**	MV, RPD, RE, and FME	[16,17,22,27,34,44,45]
Sample Entropy	**SampEn**	MV, RDP, and RE	[26,41,46,47]
Mean Amplitude	mAMP	MV, RPD, and RE	[14,15,18,23,26,27]
Mean Absolute Value	**MAV**	MV, RPD, RE, and FME	[24,29,30,31,33,35]
Mean Absolute Deviation	MAD	MC	[28]
Standard Deviation	STD	RPD, FME	[28,34]
Variance	**VAR**	MV, RE, and FME	[14,15,28,31]
Simple Square Integral	SSI	RPD and FME	[30,31]
Waveform Length	**WL**	RPD and FME	[24,28,29,31]
Willison Amplitude	**WAMP**	FME	[28,31]
Slope Sign Changes	**SSC**	MV, RPD, and FME	[24,28,30,31]
Zero Crossings	**ZC**	MV, RPD, RE, and FME	[20,24,28,31,33]
Statistical domain			
Kurtosis	**KURT**	RPD and FME	[24,28]
Skewness	**SKEW**	RPD and FME	[24,28]
Frequency domain			
Fractal Analysis	FA/FD	FME	[33,34,35]
Mean Frequency	**MNF**	MV, RPD, RE, and FME	[19,20,26,28,29,30,32,34,35,48]
Median Frequency	**MDF**	MV, RPD, and FME	[21,28,29,30,32,34,35]
The Power Spectrum Deformation Ratio	Ω	FME	[28]
Variance Of Central Frequency	VCF	FME	[30]
Total Power	**TTP**	FME	[30]

## Data Availability

The study was conducted in accordance with the Declaration of Helsinki, and the protocol was approved by the medical ethical committee of the Máxima Medical Center (Veldhoven, The Netherlands, File no: N16.074). The SOMNIA data used in this study is available from the Sleep Medicine Centre Kempenhaeghe upon reasonable request. The data can be requested by presenting a scientific research question and by fulfilling all the regulations concerning the sharing of the human data. The details of the agreement will depend on the purpose of the data request and the entity that is requesting the data (e.g., research institute or corporate). Each request will be evaluated by the Kempenhaeghe Research Board, and depending on the request, approval from an independent medical ethical committee might be required. Access to data from outside the European Union will further depend on the expected duration of the activity; due to the work required from a regulatory point of view, the data is less suitable for activities that are time-critical or require access on short notice.
